# Can wage changes solve the labour crisis in the National Health Service?

**DOI:** 10.1007/s10198-024-01737-4

**Published:** 2024-12-09

**Authors:** Xingzuo Zhou, Jolene Skordis, Junjian Yi, Yiang Li, Jonathan Clarke, Hongkun Zhang

**Affiliations:** 1 Centre for Global Health Economics, University College London, 3rd floor, 30 Guilford Street, London, WC1N 1EH UK; 2 China Center of Economic Research, National School of Development, Peking University, Beijing, 100871 China; 3 Department of Sociology, University of Chicago, 1155 E. 60th St, Chicago, IL 60637 USA; 4 Centre for Mathematics of Precision Healthcare, Department of Mathematics, Imperial College London, London, SW7 2AZ UK; 5 Department of Economics, University of Chicago, 1126 E. 59th Street, Chicago, IL 60637 USA

**Keywords:** National Health Service (NHS), Labour market, Macroeconometrical modelling, Forecasting, C53, E17, J21, J45

## Abstract

This study aimed to examine the healthcare labour demand and supply elasticity regarding wage in the National Health Service (NHS) in England amid a labour crisis. A simultaneous error-correction regression analysis was conducted using secondary data from the NHS and Office for National Statistics from 2009 Q3 to 2022 Q1. Findings indicate both labour demand and supply of HCHS doctors in the NHS are highly inelastic with respect to real wages, with only a 0.1% decrease in NHS staff hiring and a 0.8% rise in NHS staff’s willingness to work as full-time equivalents per 10% wage increase. Approximately 22% of the wage disequilibrium adjusts quarterly, indicating moderate speed of wage adjustment. Our results suggest that wage setting is not a sufficient solution to the labour crisis. Innovative and sustainable solutions are needed to reduce the demand for skilled health labour and increase the supply of health labour.

## Introduction

The coronavirus disease 2019 (COVID-19) pandemic has brought significant challenges to the global economy, including the healthcare sector [1, 2]. While lockdowns and public health interventions have made many industries vulnerable, the need for healthcare has increased globally as COVID-19 has directly and indirectly led to significant morbidity and mortality [3–5]. However, the healthcare industries in many countries struggle with staffing shortages [6, 7]. In 2023, the UK’s publicly owned health system, the National Health Service (NHS), has a staff vacancy rate of 8.9%, necessitating the recruitment of over 125,572 healthcare workers to maintain operational capacity [8]. Beyond limiting patient care capabilities, this human resource deficit imposes a psychological burden on healthcare providers [9]. This situation is further compounded by the combination of elevated COVID-19 exposure risk, high inflation rates in the surrounding economy, and stagnant real wages. This has led to a surge in wage demands from NHS workers and an increased number of employees switching to the private sector, where wage levels are more attractive, thereby, creating persistent difficulties in filling vacant posts in the public sector [10, 11]. This underscores the crucial role of government policy in resolving the ongoing healthcare workforce crisis [12].

The UK healthcare system is primarily marked by the prominence of the NHS, a tax-funded entity that provides the majority of healthcare to residents for free at the point of use. Established in 1948, the NHS was instituted to make healthcare services accessible to all irrespective of their financial capacity. It therefore holds a pivotal position in the UK healthcare structure, providing a wide spectrum of services ranging from preventive care to complex medical procedures. The NHS covers England, Scotland, Wales, and Northern Ireland, each with its own unique organisation. In this study, we focus on the NHS in England. Despite the existence of a private healthcare sector, the NHS’s influence has been dominant because of its universal accessibility, comprehensive service provision, and the inherent principles of equity upon which it was founded. According to the King’s Fund and British Medical Association (BMA), most private healthcare employees also work for NHS. Compared to 1.5 million NHS employees, approximately only 3,000 consultants work entirely in the private healthcare sector [13]. In short, unlike other countries with a significant proportion of private healthcare providers, the UK has very few private healthcare providers. As such, we reasonably consider the NHS labour market as the national healthcare labour market. Particularly, we focus on Hospital & Community Health Service (HCHS) doctors, which excludes primary healthcare staff members. Based on the determinants of the healthcare labour market discussed in micro-level studies, we propose a meso-econometric framework specifically designed to model and forecast the nuanced dynamics of wages and employment trends within the NHS.

This study focusses on the employment and wage relationship within the UK healthcare system. Previous literature has explored the causal effect of wages on employment outcomes in the micro-level market [14–16]. Particularly, Caliendo et al. (2019) find that in the short term, increased wages cause a tendency to reduce working hours. Many factors such as technology and entrepreneurship play an important role in the intricate relationship between wages and labour market demand [17–19]. In the UK healthcare market, micro-level studies have identified an inverse relationship between NHS wage levels and labour shortages, particularly in the aftermath of the pandemic, when inflation soared and real wages correspondingly diminished [20, 21]. The individual-level mechanisms behind labour inadequacy from jobs offering uncompetitive wages could be extended to understand national-level variations in the healthcare industry employment, that is, the aggregate individual-level employment outcomes form the unemployment rate. However, micro-level studies only provide the theoretical foundations and mechanisms of labour demand, labour supply, and wage elasticity. This field of study lacks a macro-level model that combines all the theories to better elucidate the interplay between employment and wages. Such a model could be used by the NHS to dynamically monitor its overall workforce and adjust its policies.

This study uses available secondary data to estimate a macro-level model of the NHS workforce to explore wage elasticities and the dynamic relationship between the unemployment rate and wages of healthcare workers in England. Several datasets are consolidated for the analyses, mainly from the NHS and the Office for National Statistics (ONS). A simultaneous equations model (SEM) that includes three equations—demand, supply, and wages—and estimates them jointly, is used [22, 23]. We incorporate an error correction mechanism (ECM) into the SEM, which allows the market to adjust towards equilibrium at different short-term adjustment speeds. We then apply this simultaneous error correction model (SECM) to our time-series data to dynamically model the relationship between wages and employment by controlling for exogenous variables suggested by micro-level studies. To the best of our knowledge, this model is the first of its kind to model the behaviour of the NHS.

Our model suggests a high inelasticity of employment regarding real wages, indicating that it is difficult for the NHS to seek new workers by increasing their wage rate. We also find that labour demand of HCHS doctors is affected by factor inputs, such as the number of hospital beds and medical supplies, which is related to labour demand of HCHS doctors through income and substitution effects. Additionally, we find that labour supply of HCHS doctors is determined by its source, that is, the number of medical graduates. Therefore, an increase in the ‘pool’ of potential labour supply will positively affect medical labour supply. Finally, we find that wage setting is not only determined by employment outcomes but is positively affected by workers’ reservation wage (i.e., the income received if they do not work for the NHS). The NHS must set higher wage rates in accordance with workers’ higher reservation wages.

We validate our findings through dynamic forecasts, that is, out-sample predictions. Four-step-ahead dynamic forecasts (approximately 10% of the data) are generated, which successfully capture the trends in employment and wage growth. Besides, main results are very close with alternative specifications. This provides assurance regarding the validity of our results, which have significant policy implications and suggest that wages, while important, are not the primary mechanism for solving the current labour crisis. Specifically, the NHS’s minimum wage rates must exceed workers’ alternative choices, but wage rates have very little effect on employment. In the short term, technological improvements that can reduce the use of medical consumables could relieve the labour crisis; in the longer term, sustainable solutions that address the labour supply directly, such as increasing investment in schooling and training, should be considered. The NHS or other analysts can update our model with additional data in the future to assist in the management of the NHS labour force, its scale, and subsidiary resources.

This study contributes to the existing literature examining the healthcare labour market. First, our study follows previous micro-level studies discussed later and combines them into a macro-level framework. To date, there is no macro-level model analysing the healthcare labour market. Our paper also complements the previous micro-level studies. Crawford et al. [24] found similar supply elasticity (0.07) in the nurse market. Our paper suggests the high inelasticity in the doctor labour market, given that doctors are more difficult to train and replace compared to nurses. We also confirm that wage setting is not a solution to solve the doctor shortage, similar to the previous conclusion that increased pay cannot solve the nurse retention problem [25]. We additionally contribute to the macro-level labour market modelling literature. Most previous literature that used a simultaneous equation model did not properly incorporate an error correction mechanism, as summarised below. This is crucial as the existence of wage resistance is modelled by the error-correction mechanism. We rigorously implement this simultaneous error-correction equation model and test its validity step-by-step.

## Background

### Determination of wages and labour outcomes: evidence from micro-level studies

According to neoclassical labour market theory, under perfect competition conditions, the supply side of the labour market is determined by the worker’s maximisation of a utility function that trades-off between leisure and income. From the employer’s demand side, labour is constantly put into production as a resource until the marginal cost of adding a worker equals the marginal productivity of an additional worker. The equilibrium wage rate and employment levels are observed where the demand and supply curves intersect [26].

Applying this theory empirically, Arestis et al. [27] use time series and panel data from nine European countries to verify that capital stock, the resource needed to produce a certain output level, is vital for determining equilibrium wage and unemployment levels. Education is also a widely discussed factor in determining the labour market. Ann [28] uses a long time series of earnings data to estimate the career technical education program outcomes regarding employment opportunities and earnings. The results show that education has a positive effect on students’ futures regarding employment outcomes and earnings. Furthermore, Schumacher [17] and Herath and Ranasinghe [29] explore the general wage and employment principle in different regions and industries and find that the labour market has a preference for skilled workers, indicating that employment opportunities for skilled workers are less affected by specific conditions.

Regarding the determination of wages, reservation wages play a significant role in wage setting. The reservation wage reflects the opportunity cost of not working and, therefore, is the minimum wage that workers accept. In other words, the reservation wage represents the gains if the worker is unemployed; if the offered wage is lower than the reservation wage, then no one will work. Thus, the reservation wage provides more bargaining power to workers when negotiating wage rates with employers. In short, reservation wages are the basis on which people build their expectations of acceptable salaries [30]. The primary reservation wage is the supplementary benefit.

In the healthcare sector, barriers to labour force entry are high due to the knowledge and skills required, leading to a more inelastic labour supply. In other words, the equilibrium in the healthcare labour market is often difficult to disrupt or adjust [31]. Focusing on the relationship between wages and employment within the healthcare sector, Steier and Moxham [32] design a model to estimate healthcare delivery. In this model, to enhance efficiency, the number of medical staff should match the health system’s capacity, as measured by the number of beds offered. This suggests a consistent relationship between the healthcare sector’s labour demand and actual healthcare delivery. Additionally, medical education is vital in expanding the labour supply to meet the demand for medical staff; Papapanou et al. [33] find that the expansion of medical education and training can be an effective solution to excess labour demand in the healthcare sector. Additionally, previous studies have found a relationship between incentives to work and the gross pension rate. Butler et al. [34] find that higher pensions lead to earlier retirement because higher expected pensions mean that older healthcare workers face less financial pressure to continue working [35, 36]. In other words, a higher pension indicates higher reservation wages.

In summary, micro-level studies have demonstrated the determinants of labour demand, labour supply, and wages. The next section explores macro-level models and how they contribute to our understanding of the relationship between wage rates and employment levels, with particular reference to the healthcare sector.

### Reviewing the literature modelling wages and employment

Many studies have proposed frameworks to model the entire economy with a component of the aggregate labour market. As summarised in Andrews [37] and reviewed by the Economic and Social Research Council, six models have been dedicated to the UK economy. All of these dedicated models have a reduced wage function. However, unlike models mainly focusing on wage setting, two models proposed by the City University of London (CUBS) and the University of Liverpool contain more integrated treatments of the labour market, as they have clear structures of labour demand, labour supply, and wage determination. Their formation of labour functions is based on a firm’s production and the utility-maximising individual choice theory. The models are relatively reliable baseline models; however, they do not fully account for the wage resistance or sluggish wage settings observed in the UK healthcare sector. While the CUBS model attempts to show a sluggish wage through the shape of error correction, it does not properly incorporate the concept of cointegration and, therefore, the ECM.

In this study, we incorporate an ECM in a simultaneous equation model (SEM) focussed on the ‘NHS labour market’. The ECM is a concept in econometrics that corrects for deviations from long-term equilibrium in a model and adjusts short-term dynamics to align with the long-term trend. This mechanism is heavily based on the principles of cointegration, where multiple series have a long-term equilibrium, as first discussed by Engle and Granger [38] and Johansen [39]. Since then, many researchers, such as Maysami and Koh [40] and Yi and Zhang [41], have implemented cointegration and ECMs in their studies. Both models use the vector error correction model, which is developed using the vector autoregressive model. However, such models usually include only common exogenous variables and the same number of lags across equations, which may not fully represent the NHS labour market in our case. As discussed in Currie et al. [42], an alternative is the SEM, which has the same functional representations under certain conditions and allows for different exogenous variables (including lag variables) across specifications. Many studies (e.g. Angrist et al. [43]), have used these structures to model demand and supply. Further, the ECM is applied in the system of simultaneous equations (Muscatelli et al., [44]; Hachicha, [45]).

## Materials and methods

### Hypothetical framework

A standard hypothetical macro-level labour market model, as in many other markets, includes a demand and supply curve:

1 $$N_{t}^{d} = a_{1} W_{t} + a_{2} Z_{t}^{d} ,$$

2 $$N_{t}^{s} = b_{1} W_{t} + b_{2} Z_{t}^{s} ,$$

where ${N}_{t}^{d}$ is the total quantity of labour demanded by firms, ${N}_{t}^{s}$ is the total quantity of labour supplied, ${W}_{t}$ is the monetary wage rate, ${Z}_{t}^{d} and {Z}_{t}^{s}$ are exogenous variables that influence labour demand and supply, respectively, and $t$ denotes the quarterly period. Normally, lagged terms, such as labour quantity and wages, are included in these exogenous terms. Furthermore, usually, ${a}_{1}<0$ and ${b}_{1}>0$, because wages are a cost on the demand side and a gain on the supply side.

At market equilibrium, that is, when ${N}_{t}^{d}{=N}_{t}^{s}={N}_{t}^{*}$ and ${{W}_{t}=W}_{t}^{*}$, we solve Eqs. (1–2) and obtain:

3 $$W_{t}^{*} = \frac{{\left( {a_{2} Z_{t}^{d} - b_{2} Z_{t}^{s} } \right)}}{{b_{1} - a_{1} }},$$

4 $$N_{t}^{*} = \frac{{a_{2} b_{1} Z_{t}^{d} - a_{1} b_{2} Z_{t}^{s} }}{{b_{1} - a_{1} }}.$$

However, in practice, labour markets usually experience deviations from the equilibrium, namely, disequilibrium, in the short term. If ${W}_{t}^{*}$ is not attained (i.e. ${{W}_{t}\ne W}_{t}^{*}$), then by re-arranging Eqs. (1–2), we obtain

5 $$W_{t} - W_{t}^{*} = \frac{{N_{t}^{s} - N_{t}^{d} }}{{b_{1} - a_{1} }}.$$

In the case of market clearing, we can obtain the wage equation in the form:

6 $$W_{t} = c_{1} Z_{t}^{d} + c_{2} Z_{t}^{s} + c_{3} Z_{t}^{w} + c_{4} ,$$

where ${Z}_{t}^{w}$ denotes wage-related exogenous variables. Many currently accepted and applied UK models are based on this baseline hypothetical framework [46]. In the case of market clearing, employment is determined by demand. However, in the absence of market clearing, the determination of employment and how real wages adjust to excess demand or supply remain unclear. To estimate wage adjustments, as proposed by Sargan (1964), we hypothesise that wages are adjusted in a pattern called an ECM. That is, any fluctuation in the wage equilibrium will impact its short-run dynamics such that

7 $$\Delta W_{t} = c_{1} \Delta W_{t}^{*} + c_{2} \left( {W_{t - 1}^{*} - W_{t - 1} } \right) + c_{3} Z_{t}^{w} ,$$

where ${Z}_{t}^{w}$ denotes a vector containing all the age-related variables. In practice, wages are not adjusted immediately, but quarterly or annually. The partial adjustment model is a commonly used pattern that is preferred because it accounts for wage resistance. By imposing the assumption that ${\text{c}}_{1}={\text{c}}_{2}$, Eq. (7) becomes a partial adjustment model

8 $$\Delta W_{t} = c_{1} \left( {W_{t - 1}^{*} - W_{t - 1} } \right) + c_{3} Z_{t}^{w} .$$

Provided ${\text{c}}_{1}>0$, an increase in ${\text{c}}_{1}$ leads to a higher speed of wage adjustment towards equilibrium. If ${\text{c}}_{1}=1$, then the market clears instantly.

Combining Eqs. (5) and (7), we attain

9 $$\Delta W_{t} = \frac{{c_{3} Z_{t}^{w} }}{{1 - {\text{c}}_{1} }} - \frac{{b_{1} \left( {N_{t}^{s} - N_{t}^{d} } \right)}}{{\left( {{\text{b}}_{1} - a_{1} } \right)\left( {1 - b_{1} } \right)}}.$$

To estimate the wage equation, we substitute Eqs. (1) and (2) into (9) to obtain the reduced-form equation [47]:

10 $$\Delta W_{t} = d_{1} W_{t - 1} + d_{2} Z_{t}^{d} + d_{3} Z_{t}^{s} + d_{4} Z_{t}^{w} .$$

The error correction functions of labour demand and supply follow similar structures.

In summary, the aggregate views of the operation of the labour market are characterised by the speed at which the real wage moves to clear the market. Since our setting has a wage specification with an error-correction term, our view of operations of the HCHS doctor market is that the market does not clear. In other words, the market is in disequilibrium. Our model has similar settings compared to some other models that are still maintained, updated and implemented for the aggregate economy. These models are designed for the whole economy, including both the capital and labour market. Still, the modelling settings for the labour market are somehow similar. Such models include the His Majesty’s Treasury (HMT) model, now maintained by the Office for Budget Responsibility (OBR) [48], the London Business School (LBS) model, later acquired and maintained by the Bank of England [49].

### Determinants of labour demand

The NHS is a non-profit organisation funded by hypothetical and unhypothetical taxation. Its objective is to attain universal health coverage for all UK residents [50], and it ‘will be devoted solely to the benefit of the people that the NHS serves’ [51]. Unlike a traditional company that exhibits profit-maximisation behaviour, the NHS aims to provide healthcare services to as many people as possible, given finite resources. In short, labour demand is mainly determined by the population’s demand for healthcare and, therefore, related to the capital stock needed to satisfy the demand for healthcare services, which is the capital stock argument [27]. There are several related proxies, such as the number of beds owned by the NHS to meet Britain’s demand for healthcare [52].

The main cost of labour is wages. A higher cost of labour should lead to a lower demand for labour through the income effect. Thus, we consider nominal wages and employer pension contributions in this study. We normalise the wage with the price index in the healthcare industry (‘NHS’ health index) as it relates to NHS expenses specifically. Similar to wages paid regularly, other variable costs such as those of medical supplies and utility bills should be considered. Conventionally, if an increase in the price of other inputs leads to an increase in labour demand, the substitution effect dominates (i.e. substitutes). If an increase in the price of other inputs leads to a decrease in labour demand, the income effect dominates (i.e. complements). In this study, we consider the prices of energy (utility bills) and medical supplies.

### Determinants of labour supply

Individuals’ employment decisions are primarily determined by the real wage they receive [53], that is, the wage minus the pay-as-you-earn income tax and employees’ pension contributions. As the UK healthcare labour market is dominated by the NHS, medical students are mostly hired by the NHS once they graduate; therefore, the number of medical graduates has a direct influence on the medical labour supply. Additionally, fluctuations in the working-age population should have a direct effect on labour supply. Assuming constant rates of new joiners to the working-age population, a decrease in the working-age population could be due to more retirement, creating a lower labour supply. Furthermore, labour supply is related to the unemployment rate [54]. Gustman and Steinmeier [55] find that a lower employment rate encourage youth to join the labour market. Particularly within the NHS, the source of unemployment mainly stems from frictional and structural unemployment among the youth. Frictional unemployment includes cases such as new graduates moving from colleges or training institutes to hospitals, and structural unemployment includes cases such as new graduates don’t have the essential skills and experiences for the vacancies. Additionally, the NHS is increasingly relying on immigrants, particularly those from the Global South, to fill the vacancies. Overseas staff now accounts for a significant portion of total active workforce.

### Wage determinants

The main driver of wages is the unemployment rate. Higher unemployment rates indicate a more competitive labour market; therefore, employees’ bargaining power is weaker, leading to lower wages. The bargaining power argument also supports the idea that as the reservation wage (i.e. supplementary benefits) increases, wage levels should also increase. Wages are further determined by the gross pension replacement rate, ‘the level of pensions in retirement relative to earnings when working,’ as defined by the Organisation for Economic Co-operation and Development [56]. Higher gross pension replacement rates indicate higher income after retirement, leading workers to leave the labour market. This can also be viewed as a reservation wage after retirement [57]. Higher reservation wages after retirement cause workers to be more willing to leave the labour market and less willing to re-enter the labour market. Studies also show that this positive relationship holds in the 3rd to 5th quintile of income, which is the salary of the NHS staff [58].

### Data sources

The data used in this study are time-series data from 2009 Q3 to 2022 Q1. This study mainly uses datasets provided by the NHS England, including NHS Workforce Statistics (NHS-WS), NHS Staff Earnings Estimates (NHS-SEE), and NHS Bed Availability and Occupancy Data (NHS-BAO). The NHS-WS is a monthly updated series that records the number of people working in the NHS England. The NHS-SEE is a monthly data series published by the NHS Digital that illustrates the mean annual earnings of the NHS England workforce. The NHS-BAO is collected quarterly by the NHS England. It collects the total number of available-bed days and the total number of occupied-bed days.

We also consolidate several other datasets from the ONS, UK government (GOV-UK), OECD, World Bank, and BMA, which report economic statistics, the number of medical graduates, unemployment rates in the healthcare sector, price levels, tax rates, and pension contribution rates. Appendix Table 5 presents the specific data extracted and derived from the different sources. Table 1 provides a summary of the descriptive statistics, and the changes in the price indices are available in Appendix Figure 2.

**Table 1:** Summary of statistics

Variable	Obs	Mean	Min	Max
Quarter	51		2009Q3	2022Q1
NHS HCHS doctors (FTE)	51	106851	94538.50	128391.80
NHS HCHS doctors (headcount)	51	113942.70	101445	136597
Unemployment rate (Health)	51	2.77%	1.90%	3.95%
Mean annual basic pay (real terms in 2022 Q1 £)	51	70773.74	1223.213	66317.23
Number of beds	51	133560.80	118473.40	158461.10
Employer’s rate of pension contributions	51	15.68%	14.00%	20.68%
Employee’s rate of pension contributions	51	5%	5%	5%
Working age population (000 s)	51	34389.21	33621.34	34933.08
Medical graduates	51	8655.10	8210	9140
NHS HCHS overseas doctors (headcount)	51	20268.76	16452	31858
Basic rate of tax	51	20%	20%	20%
Supplementary benefit	51	70.37	59.83	77.28
Gross pension replacement rate(%)	51	30.09	21.60	49.00

### Labour market outcomes

The private healthcare labour market is negligible in the UK. Thus, we treat the healthcare labour market as public (i.e. the NHS labour market). The illustration of the number of staff (full-time equivalents [FTE]) hired by the NHS throughout the 51 quarters is available in Appendix Figure 3. Over the past decade, the number of staff (FTE) hired by the NHS has increased by approximately 35%. As the labour market is determined by the demand side [37], we use the current number of FTE staff hired as the labour demand of the NHS.

Because almost all healthcare workers are hired by the NHS, we reasonably treat the number of doctors registered in the NHS as the maximum possible labour supply. The illustration of the number of NHS HCHS doctors (headcount) is also available in Appendix Figure 3. The implicit unemployment rate (i.e. the difference between the FTE and headcount) has been approximately 6% in the past decades (Appendix Figure 4).

Another significant labour market variable is the average wage. For this purpose, we use the mean annual basic pay, which increased by approximately 24% in nominal terms over 13 years of our study range. For our analyses, we calculate real wages using the GDP deflator because real wages are offset by inflated expenses in all sectors. The real wages (2022 Q1 £) decreased consistently until the pandemic and recovered thereafter. The illustration is available in Appendix Figure 5.

### Empirical estimation

Based on the standard hypothetical model discussed, the labour demand function should be determined by productivity. In the labour market of health professionals in England, the NHS dominates labour demand. Unlike private firms, which aim to maximise profits, the NHS is a public healthcare system funded by taxpayers. As such, the labour demand of ‘public’ health professionals in England is determined by the patients requiring treatment under the NHS in England. In the standard format, we rewrite Eq. (1) as

11 $$N^{d} = D\left( {C,P^{e} ,\frac{{W\left( {1 + e} \right)}}{{P^{h} }},P^{m} } \right),$$

where ${N}^{d}$ is the labour demand (number of doctors in FTE), $C$ denotes the capital stock held by the NHS to treat patients (we use the number of beds as a proxy), ${P}^{e}$ denotes the price of energy (domestic energy price indices), ${P}^{m}$ denotes the price of materials (price index of medical supplies), $e$ is the employer’s rate of National Insurance contributions, and ${P}^{h}$ denotes the price index in the healthcare industry (NHS).

A generic three-stage least squares (3SLS) model should also include labour supply and wage specifications. However, labour outcomes are determined by the demand side and any excess demand or supply will result in labour ‘temporarily’ working or involuntarily unemployed. Based on the hypothetical model, a typical labour supply specification is

12 $$N^{s} = S\left( {MG,\frac{{W\left( {1 - t - n} \right)}}{P},u, POP, OD} \right),$$

where ${N}^{s}$ denotes the labour supply (number of doctors in the headcount); $MG$ denotes the number of medical graduates; $W$ denotes the nominal wage rate; $t and n$ denote the basic rate of tax and employee contribution towards the pension scheme, respectively; $P$ denotes the GDP deflator; $u$ denotes the unemployment rate in the health industry; $POP$ denotes the population; and $OD$ denotes the supply of doctors from rest of the world. Additional lags of the variables are added as companions.

Finally, the wage equation can be derived from Eq. (6). In accordance with Eq. (6), we estimate the equilibrium wage using all control variables, which generates the reduced-form wage equations:

13 $$\frac{W}{P} = W\left( {u,\frac{SB}{P},RR} \right),$$

where $SB$ denotes the supplementary benefit level, and $RR$ denotes the replacement rate. We follow the same process of variable transformations as that in the literature, for example, taking logs [54].

Owing to factors such as wage resistance, the market may not adjust or clear instantaneously. That is, disequilibrium may persist in the short term. In such cases, the ECM has been widely discussed and applied to dynamic forecasting [47]. In ECM, cointegration plays an important role as it separates long-term relationships and short-term movements towards it. Similar to Johansen (1995) and Enders’ (2008) standard method of cointegration analysis, the system of Eqs. (11–13) becomes^1^

14 $$\Delta Y_{t} = c + \mathop \sum \limits_{i = 1}^{k} \Gamma_{1i} \Delta Y_{t - i} + \mathop \sum \limits_{i = 0}^{k} \Gamma_{2i} \Delta Z_{t - i} + \Theta \widehat{EC}_{t - 1} + e_{t} ,$$

where.

$$Y_{t} = \left( {\ln N_{t}^{d} ,\ln N_{t}^{s} ,\ln \left( \frac{W}{P} \right)_{t} } \right);$$

$$Z_{t} = \left( {\ln C_{t} ,\ln \left( {\frac{{P^{e} }}{{P^{h} }}} \right)_{t} ,\ln \left( {\frac{{P^{m} }}{{P^{h} }}} \right)_{t} ,\ln \left( {\frac{{W\left( {1 + e} \right)}}{{P^{h} }}} \right)_{t} ,\ln MG_{t} ,\ln POP_{t} ,\ln OD_{t} , \ln \left( \frac{W}{P} \right)_{t} ,\ln \left( {1 + u_{t} } \right),\ln \left( \frac{SB}{P} \right)_{t} ,\ln RR_{t} } \right);$$

$$\widehat{EC}_{t - 1} = \left( {\widehat{{u_{t - 1}^{d} }},\widehat{{u_{t - 1}^{s} }},\widehat{{u_{t - 1}^{w} }}} \right).$$

${\widehat{EC}}_{t-1}$ Is a vector of error correction terms derived from the predicted residuals of the cointegration regressions. The estimates in Eq. (15–17) are also long-term effects and elasticities:

15 $$\ln N^{d} = \hat{\alpha }_{0} + \hat{\alpha }_{1} \ln C + \hat{\alpha }_{2} \ln \left( {\frac{{P^{e} }}{{P^{h} }}} \right) + \hat{\alpha }_{3} \ln \left( {\frac{{P^{m} }}{{P^{h} }}} \right) + \hat{\alpha }_{4} \ln \left( {\frac{{W\left( {1 + e} \right)}}{{P^{h} }}} \right) + u_{t - 1}^{d} ,$$

16 $$\ln N^{s} = \hat{\beta }_{0} + \hat{\beta }_{1} \ln MG + \hat{\beta }_{2} \ln \left( \frac{W}{P} \right) + \hat{\beta }_{3} \ln \left( {1 + u} \right) + \hat{\beta }_{4} \ln POP + \hat{\beta }_{5} \ln OD + u_{t - 1}^{s} ,$$

17 $$\ln \frac{W}{P} = \hat{\gamma }_{0} + \hat{\gamma }_{1} u + \hat{\gamma }_{2} \ln \left( \frac{SB}{P} \right) + \hat{\gamma }_{3} {\text{ ln}}RR + u_{t - 1}^{w} .$$

## Results

Before selecting the models, we test for cointegration. There are two tests for cointegration generally accepted in the literature, proposed by Engle and Granger (1987) and Johansen (1988). We implement the Engle-Granger test because the Johnson test relies on asymptotic properties and may produce unreliable results when the sample size is not sufficiently large. The Engle-Granger approach comprises two steps. The first step is to check whether all variables in the equation are integrated at order 1 (i.e. the I(1) process). To verify this, we test the stationarity of the variables. If a variable is not stationary, we must difference it and test the stationarity of the differenced variables again. If a variable has an order of integration of 1, then it is stationary after the first differencing. The Phillips–Perron (PP) and modified Dickey-Fuller (DF) unit root tests can be applied to confirm stationarity, and the null hypothesis is the existence of a unit root. If the null hypothesis is rejected, stationarity is satisfied. Table 2 shows the PP and modified DF unit root test results. The statistics show evidence that all series are I(1) processes that satisfy the preliminary process before investigating the cointegration relationships.

**Table 2:** PP and DF unit root tests

Variable	Test statistic (PP)	Test statistic (DF)
$\ln N^{d}$	2.334 −9.479***	−1.225 −9.239***
$\ln N^{s}$	2.595 −8.990***	−1.285 −7.739***
$\ln C$	−3.211** −7.355***	−1.583 −6.653***
$\ln \left( {P^{e} } \right)$	−0.379 −8.274***	−2.618 −4.816 ***
$\ln \left( {P^{m} } \right)$	−0.421 −9.610***	−2.798* −4.707***
$\ln \frac{{W\left( {1 + e} \right)}}{{P^{h} }}$	−0.244 −7.040***	−1.543 −4.745 ***
$\ln MG$	−2.598 −7.483***	−2.878 * −3.528**
$\ln \left( {1 + u} \right)$	−1.294 −9.495***	−1.684 −4.626***
$\ln POP$	−1.956 −4.345***	−3.528** −3.052**
$\ln OD$	−2.052 −4.015***	−2.650 −3.619**
$\ln \frac{W}{P}$	−2.834* −10.198***	−2.635 −5.417***
$u$	−1.295 −9.482***	−1.683 −4.625***
$\ln \left( \frac{SB}{P} \right)$	−2.031 −8.827***	−1.473 −5.012
$\ln RR$	−1.461 −6.770***	−1.606 −4.550***

Each first row illustrates the statistics of the I(0) process, and the second row illustrates the statistics of the I(1) process; lags are chosen based on the Newey-West (PP) and Schwert criteria (DF), respectively

*p < 0.10, **p < 0.05, ***p < 0.01

In the second step of the Engle-Granger approach, if the residuals of the static cointegration regressions (15 ≠ 17) are stationary, the series are cointegrated. We estimate three cointegration equations using the fully modified ordinary least squares method, as it corrects for both endogeneity and serial correlation [59, 60]. Table 3 shows the results of the Engle-Granger test. There is strong evidence of cointegration; therefore, ECMs should be applied.

**Table 3:** Engle-Granger test

Variable	Test statistic	Conclusion
$u_{t - 1}^{d}$	−2.414***	Cointegrated
$u_{t - 1}^{s}$	−4.604***	Cointegrated
$u_{t - 1}^{w}$	−3.633***	Cointegrated

*p < 0.10, **p < 0.05, ***p < 0.01

We then estimate the equation system as shown in Eq. (14). Instead of estimating the three equations separately, we jointly estimate them using 3SLS, as this addresses the issue of endogeneity. To choose the best model specification, that is, the number of lag variables, we use the Akaike information criterion (AIC) and Bayesian information criterion (BIC), which analyse the model fit while controlling the size of the model, that is, the number of parameters. The model with the smallest AIC/BIC is preferred because it provides more accurate forecasts while minimising the size of the model.

We also test the serial correlation of the model residuals. Our dynamic forecasts may be misspecified if the residuals are serially correlated, that is, they are not white-noise series. If our best selected model according to the AIC/BIC criteria is misspecified, for example, overfitting the model, we then test our second best selected model. We conduct the Ljung-Box portmanteau (Q) test, which has a null hypothesis positing that the residual is white noise, that is, serially uncorrelated. Table 4 presents these two tests of the best selected models, which do not indicate problems of model misspecification.

**Table 4:** Residual serial correlation tests

Information criteria	Test statistic	Specification
AIC = −993.1498 BIC = −954.9210	27.53* (0.09)	Demand
	21.48 (0.31)	Supply
	11.93 (0.92)	Wage

*p < 0.10, **p < 0.05, ***p < *0.01*, p-values reported in parentheses

The model terms of our best selected model are Eqs. (18–20):

18 $${\Delta {\text{ ln}}N_{t}^{d} } = \mathop{ {0.81\Delta {\text{ ln}}N_{t - 4}^{d} }}_{(13.53)} - \mathop{{0.09 \Delta \ln C_{t} }}_{( - 2.38)} + \mathop{{0.03 \Delta \ln P_{t - 1}^{e} }}_{(1.50)} + \mathop{ {0.01\Delta \ln P_{t - 4}^{h} }}_{(0.06)} - \mathop{ {0.01 \Delta \ln \frac{{W_{t - 3} \left( {1 + e} \right)}}{{P_{t - 3}^{h} { }}}} }_{( - 0.07)} - \mathop{{0.09{ }\widehat{{u_{t - 1}^{d} }}}}_{( - 2.35)} + \mathop{ {0.00}}_{(0.55),}$$

19 $${\Delta {\text{ ln}}N_{t}^{s} } = \mathop{{0.11\Delta {\text{ ln}}N_{t - 3}^{s} }}_{(1.45)} + \mathop{{0.87\Delta {\text{ ln}}N_{t - 4}^{s} } }_{(9.91)} + \mathop{{0.08{ }\Delta \ln \frac{{W_{t - 3} }}{{P_{t - 3} }}}}_{(1.09)} + \mathop{{0.40\Delta \ln POP_{t - 1} }}_{(0.34)} + \mathop{{0.08\Delta \ln \left( {1 + u_{t} } \right)}}_{(0.29)} + \mathop{{0.01\Delta \ln MG_{t - 1} }}_{(0.29)} + \mathop{{0.02\Delta \ln OD_{t - 4} }}_{(0.51)} - \mathop{{0.02 \widehat{{u_{t - 1}^{s} }}}}_{( - 0.35)} + \mathop{{0.00}}_{(0.35),}$$

20 $${\Delta \ln \frac{{W_{t} }}{{P_{t} }}} = \mathop{{ - 0.29 \Delta \ln \frac{{W_{t - 2} }}{{P_{t - 2} }}}}_{( - 3.10)} - \mathop{{0.64 \Delta u_{t - 1} }}_{( - 1.51)} + \mathop{{0.34 \Delta \ln \frac{{SB_{t} }}{{P_{t} }}}}_{(7.30)} + \mathop{{0.01\Delta \ln RR_{t} }}_{(1.36)} - \mathop{{0.22 \widehat{{u_{t - 1}^{s} }}}}_{( - 2.36)} - \mathop{{0.00}}_{( - 1.66).}$$

## Discussion

The above specifications are consistent with our arguments regarding the determinants of labour demand, labour supply, and wages. In the demand specification, we observe that an increase in the number of beds is associated with lower labour demand. In the short run, NHS needs to address the substitution effect within its fixed budget constraints. Particularly, a critical issue that currently exists in the NHS is underspend [61–64]. Although the Department of Health and Social Care’s budget has been increasing since the last decade, the demand for healthcare also increased and a £362 billion underspend was accumulated since 2009/10. UK’s current health expenditure per capita in real terms was constant in the past decade, and with a constant budget, if more funds are allocated towards increasing bed capacity, there will be a corresponding decrease in labour hiring to maintain financial balance. To address this issue, the NHS announced a Long-Term Workforce Plan, with a significantly higher amount of funding set in the next decade. This plan aims to directly increase the hiring budget for healthcare staff such that the income effect can dominate the substitution effect. Regarding consumables (i.e. medical supplies and energy), an increase in their prices leads to higher labour demand, indicating dominant income effects. This implies that consumables and doctor demand are complementary. Specifically, an increase in the number of beds and staff results in higher consumption of medical equipment. Further, higher wages discourage the NHS from employing more staff as wages are the ‘price’ of labour.

In the supply specification, the working-age population and overseas recruitment positively affect labour supply of NHS HCHS doctors. We further find that an increase in the unemployment rate is associated with a higher labour supply. When new workers enter the labour market, the labour supply and the unemployment rate increase until new workers find a job. This mechanism dominates the discouraging effects of the unemployment rate on labour supply in the very short run. An increase in the number of medical graduates implies a higher labour supply, but the association is very low, given the fact that medical students need extensive training before entering the market. However, none of these estimates are statistically significant at the 5% level. This could be due to the small sample size, which is a common issue in time-series analyses.

In the wage specification, the unemployment rate negatively affects real wages, as expected. The coefficients of supplementary benefits and gross pension replacement rates in the wage specification are positive, which fits the reservation wage argument. Further, according to our model, employment (both labour demand and supply) is highly inelastic regarding real wages. The estimate of real wage in the demand specification represents the elasticity of wage: A 1% increase in real wage only induces 0.01% less labour demand or 0.08% more labour supply. This corresponds to the following reality: (1) The healthcare services provided by doctors are extremely difficult to replace with other factors of production; (2) Because the entry barrier to medical careers is high due to the required education level, it is difficult to substitute medical staff; (3) The extensive learning and training usually takes at least seven years to complete; (4) Labour supply is difficult to stimulate by an increase in wages, indicating an irreplaceable and constrained doctor supply in the short term.

The adjustment parameters for the error correction terms are as expected. All three adjustment parameters are negative (Demand: −0.09, Supply: −0.02, Wage: −0.22). This is because, if the wage is above the long-term equilibrium, we would expect wages to decrease back towards the equilibrium (and vice versa). Hence, the negative signs suggest that the system is stable and returns to equilibrium after a shock, with some lags. The speed of wage adjustment is slow; approximately 22% of the difference between the current real wage and the long-term real wage equilibrium is adjusted in the following quarters, indicating strong evidence of wage resistance. Notably, like many other institutions, the NHS regularly publishes its wage adjustments.

Furthermore, the small coefficients of the error correction terms in the labour specifications may imply that employment (labour demand) and labour supply adjust slowly when there is a shock, as pre-assessments and training are necessary. Short-term dynamics are not strongly linked to the long-term equilibrium, as it is only possible to prepare a limited number of medical staff within a short time. In other words, there is very little short-term correction; that is, the system adjusts poorly in the short term to return to its long-term equilibrium after a shock or disturbance.

### Validating the results

**Fig. 1:**
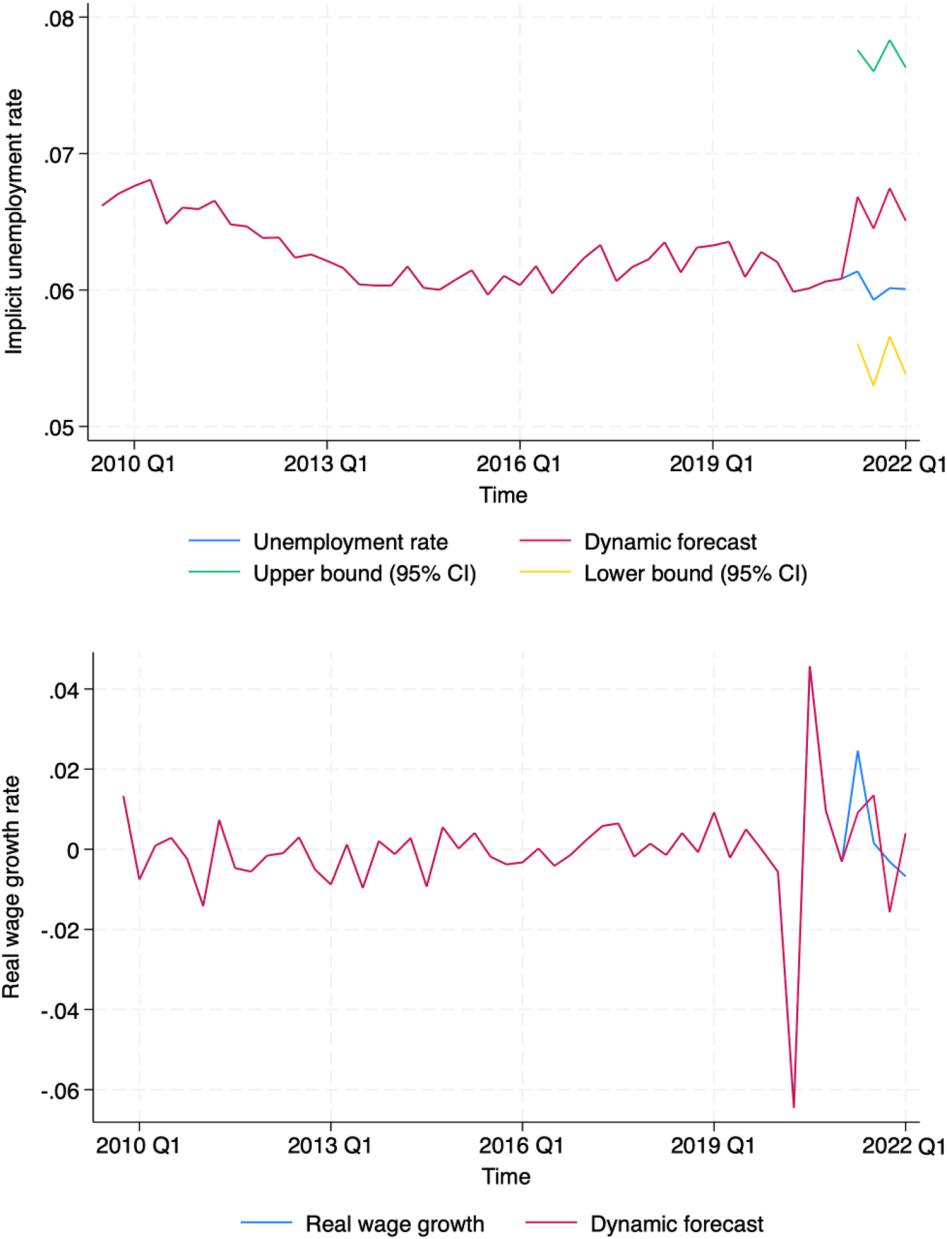
Unemployment rate and real wage growth rate forecasts. *CI* confidence interval

Figure 1 presents our dynamic forecasts of the implicit unemployment rate and our four-step ahead forecasts of real wage growth rates. Our dynamic forecasts are similar to the actual observed values and capture the trend; this indicates the reliability of our results for policymakers and the NHS regarding forecasting future healthcare labour supply, demand, and equilibrium wages in England. As forecasts are produced dynamically (i.e. out-of-sample forecasts), the robustness of our model is supported.

We further validate our results using alternative models with different number of lags. Table 5 presents the elasticities, adjustment parameters and their information criteria. Compared to our best selected model, elasticities and wage resistance are very close, indicating that the robustness of our model is supported.

**Table 5:** Elasticities and wage resistance with alternative models

	Main	(2)	(3)	(4)
Information criteria	AIC = −993.1498 BIC = −954.9210	AIC = −970.9370 BIC = −930.9706	AIC = −967.6513 BIC = −920.7342	AIC = −962.8626 BIC = −910.7325
Demand equation				
Choice of AR lags	4	3,4	1,2,3,4	1,2,3,4
Demand elasticity	−0.01	−0.01	−0.00	−0.00
Supply equation				
Choice of AR lags	3,4	3,4	1,2,3,4	1,2,3,4
Supply elasticity	0.08	0.11	0.12	0.11
Wage equation				
Choice of AR lags	2	2	2	1,2,3,4
Wage resistance	−0.22	−0.23	−0.23	−0.23

## Conclusions

In this study, we established a new framework to analyse, model, and forecast labour market outcomes in the UK’s healthcare sector, particularly in England. Through an SECM that includes several exogenous controls, we find that labour demand and supply are highly inelastic regarding real wages and, to some extent, wage resistance; this indicates the difficulty of hiring more staff to expand the scale of delivery and meet the increasing demand for healthcare. Currently, NHS England has long waiting times for patients and understaffed hospitals; therefore, a more sustainable healthcare system is needed. Wage setting is not a fundamental issue in the current healthcare market. Instead, the key issues are constraints on labour supply.

The current healthcare system relies heavily on immigrants to fill vacancies [65], which is unlikely to be sustainable and is often considered unethical [66]. Instead, policymakers and the NHS should consider increasing expenditures on medical education in both the UK and the Global South. This would increase the ‘pool’ of medical labour supply domestically and globally without controversially over-recruiting from settings where the healthcare workforce is often even more depleted. However, this policy will not yield short-term results, and NHS’s reliance on the inward migration of skilled workers is likely to persist.

As the current healthcare workforce ages or moves to the private sector, according to the increasing trend [67], shortages in labour supply are likely to be exacerbated. Innovative and sustainable solutions will need to be identified in the short term to reduce the demand for skilled healthcare labour and increase the healthcare labour supply. Particularly, technological improvements, such as the development of AI in healthcare may offer opportunities to increase productivity, and therefore, might also reduce the demand for specialised doctors, as mentioned in the NHS long-term plans. However, more robust evidence is needed to assess the likely extent of this technology effect—if any. Moving patients from the public to private sector will not resolve the shortage of labour supply in a closed system and can result in cost escalation.

This study has some limitations. First, non-financial factors, such as job quality and staff satisfaction that affect the labour supply, are not included in our model due to the lack of a robust and consistent measure. Particularly, labour supply is determined by the utility gained from working. Utility can be described as satisfaction or benefits, and wage is a main driving force of it. Another driving force could be staff satisfaction and job quality in the NHS. Currently, there is a concerning level of unhappiness among the NHS staff according to the NHS Staff Survey [68], which would negatively influence retention, and therefore, labour supply. The NHS Staff Survey evaluates staff satisfaction and provides staff experience scores in multiple dimensions, such as inclusivity, recognition, burnout, flexibility, safety and health, etc. These scores cover most aspects of staff satisfaction, so they are relatively robust measures of staff satisfaction. Ideally, these factors should be considered and added to our model. However, these measures are only available after a significant change to the survey in 2021. In addition to considering inward migration, our current model does not account for outward migration due to the lack of relevant data. Furthermore, while our model could benefit from further decomposition of specialties, we currently only disaggregate the overall healthcare labour market to focus on HCHS doctors due to data limitations. Moreover, as in many other macroeconometric modelling studies, the relatively small sample size is a limitation, as some results are not highly significant. Nevertheless, our sample size was 51, and our specifications were correctly specified, as tested. In the future, policymakers and the NHS can continuously update the model, and consequently, obtain more asymptotically precise results.

## Figures and Tables

**Fig. 2:**
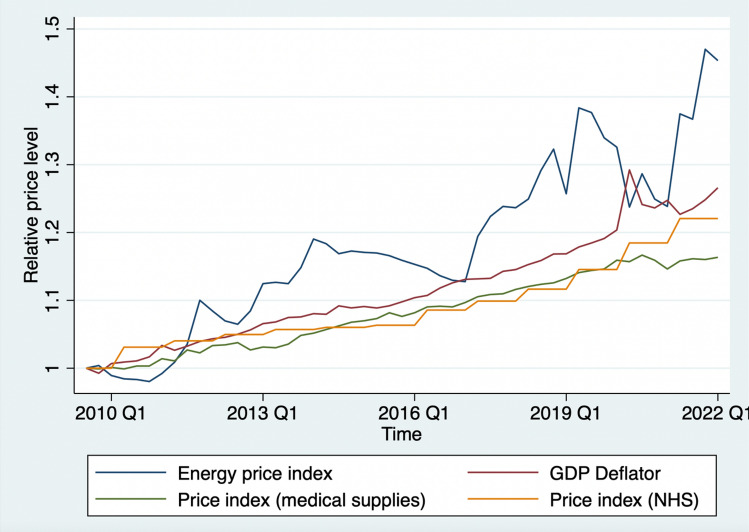
Price indices (Base = 2010 Q1). *GDP* Gross Domestic Product, *NHS* National Health Service

**Fig. 3:**
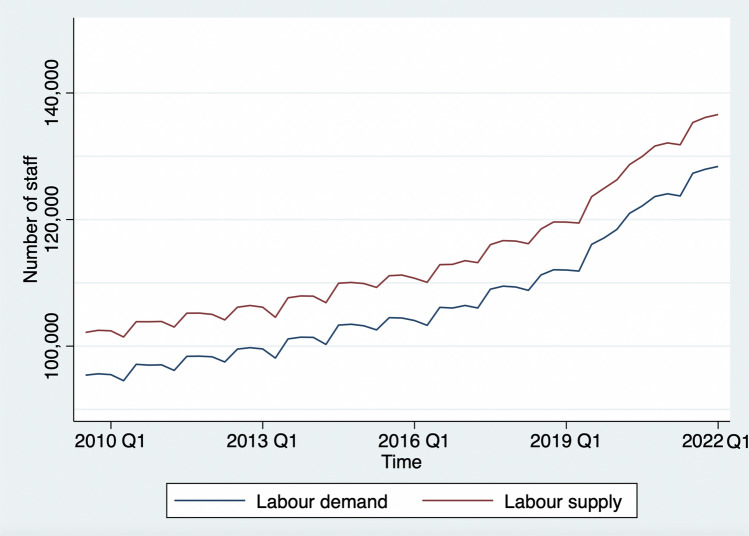
Labour demand and supply of the National Health Service

**Fig. 4:**
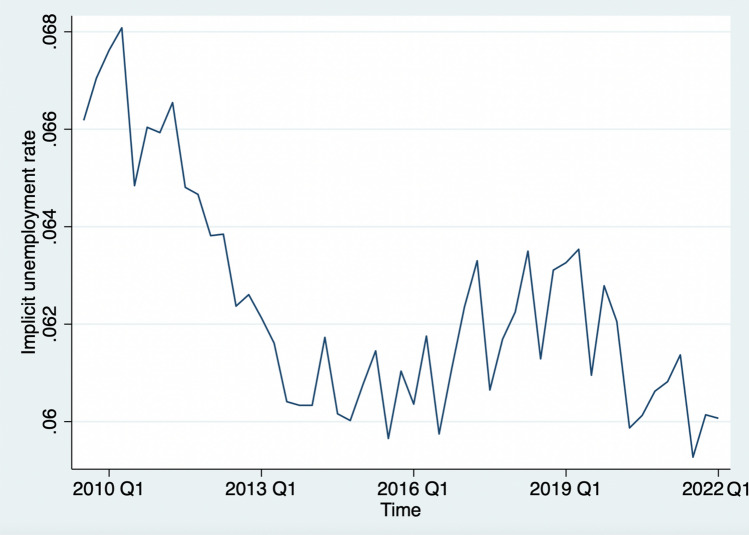
Implicit unemployment rate

**Fig. 5:**
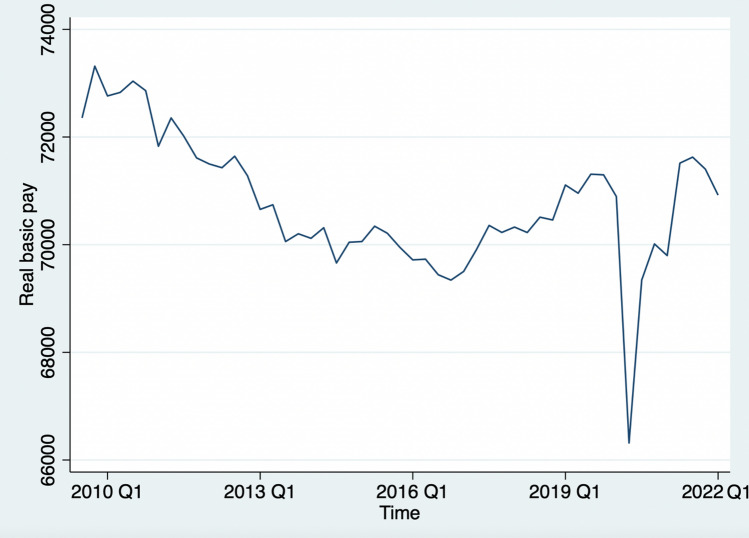
Real wage (£) over the study period (2022 Q1 £)

## Data Availability

Data is available upon request.

## Appendix

See Table 6 and Figures. 2, 3, 4, 5

**Table 6:** List of data sources

Variables	Sources	Notes
Number of NHS employees (FTE)	NHS-WS	Number of NHS HCHS doctors in Full Time Equivalence
Number of NHS labour force (Headcount)	NHS-WS	Number of NHS HCHS doctors in Headcount
Unemployment rates in health sector (U)	ONS	We used human health & social work activities in ONS-UNEM03: Unemployment by previous industrial sector
Mean annual basic pay	NHS-SEE	
Number of available beds	NHS-BAO	
Domestic energy price index	GOV-UK	
Population	ONS	
Medical products, appliances and equipment price index	ONS	
NHS price index	NHS	
Employer’s rate of pension contributions	BMA	
Employee’s rate of pension contributions	BMA	
Medical graduates	OECD	
Basic rate of tax	GOV-UK	
Implied GDP deflator at market prices: SA Index	ONS	
Supplementary benefit	GOV-UK	
Replacement rate	OECD	

## Footnotes

1 $\frac{W}{P}$ is used instead of $\frac{{W\left( {1 - t - n} \right)}}{P}$ as *t* and *n* do not change within the timeframe.
